# Assessment of Seven Clinical Tumor Markers in Diagnosis of Non-Small-Cell Lung Cancer

**DOI:** 10.1155/2018/9845123

**Published:** 2018-12-11

**Authors:** Zhong-qing Chen, Ling-sha Huang, Bo Zhu

**Affiliations:** Department of Clinical Laboratory, Guangxi Medical University Affiliated Tumor Hospital, Nanning, 530021 Guangxi Province, China

## Abstract

**Background:**

The correlation between tumor markers (TM) and TNM stage of non-small-cell lung cancer (NSCLC) has not been widely reported.

**Methods:**

TM levels (CEA, CA125, CA15-3, CA19-9, CA72-4, CYFRA21-1, and SCC-Ag) in 424 cases of lung adenocarcinoma (LAC), 166 cases of lung squamous cell carcinoma (LSCC), and 103 cases of benign chest disease (BCD) were analyzed before treatment.

**Results:**

By *Kendall's tau-b* correlation analysis, CEA, CA125, CA15-3, CA19-9, CA72-4, CYFRA21-1, and SCC-Ag were correlated with T stage of LAC (*r* = 0.235, *p* < 0.05; *r* = 0.298, *p* < 0.05; *r* = 0.254, *p* < 0.05; *r* = 0.063, *p* < 0.05; *r* = 0.080, *p* < 0.05; *r* = 0.268, *p* < 0.05; and *r* = 0.080, *p* < 0.05). CEA, CA125, CA15-3, CA19-9, CA72-4, and CYFRA21-1 were correlated with N stage of LAC (*r* = 0.356, *p* < 0.05; *r* = 0.415, *p* < 0.05; *r* = 0.340, *p* < 0.05; *r* = 0.117, *p* < 0.05; *r* = 0.175, *p* < 0.05; and *r* = 0.351, *p* < 0.05). CEA, CA125, CA15-3, CA19-9, CA72-4, and CYFRA21-1 were correlated with M stage of LAC (*r* = 0.365, *p* < 0.05; *r* = 0.353, *p* < 0.05; *r* = 0.293, *p* < 0.05; *r* = 0.135, *p* < 0.05; *r* = 0.169, *p* < 0.05; and *r* = 0.312, *p* < 0.05). CA125, CYFRA21-1, and SCC-Ag were correlated with T stage of LSCC (*r* = 0.202, *p* < 0.05; *r* = 0.233, *p* < 0.05; and *r* = 0.099, *p* < 0.05). CA125 and CYFRA21-1 were correlated with N stage of LSCC (*r* = 0.178, *p* < 0.05 and *r* = 0.284, *p* < 0.05). CA125, CA15-3, and CYFRA21-1 were correlated with M stage of LSCC (*r* = 0.214, *p* < 0.05; *r* = 0.152, *p* < 0.05; and *r* = 0.213, *p* < 0.05). Combining hazard ratio, AUC, sensitivity, specificity, NPV, and PPV, it was concluded that CEA and CYFRA21-1were the most related TM of LAC. SCC-Ag and CYFRA21-1 were the most related TM of LSCC.

**Conclusions:**

CEA combined with CYFRA21-1 contributed to auxiliary diagnosis of LAC. CYFRA21-1 combined with SCC-Ag contributed to auxiliary diagnosis of LSCC. CEA, CA125, CA15-3, CA19-9, CA72-4, and CYFRA21-1 were correlated with primary tissue and metastasis of LAC. CA125 and CYFRA21-1 were correlated with primary tissue and metastasis of LSCC.

## 1. Introduction

Data released by the National Cancer Registry (NCCR) of China in 2018–2015 showed that primary lung cancer was the first cancer in morbidity and mortality for four consecutive years [1–3]. The diagnosis of early-stage non-small-cell lung cancer (NSCLC) was the first step toward successful clinical therapy and increased patient survival. Making full use of the existing means to diagnose early NSCLC has been concerned. Histopathological examination is the gold standard for the diagnosis of NSCLC. However, due to the invasiveness of histopathological examination, chest computed tomography and tumor markers (TM) test preceded histopathological examination. The TM test results were increasingly influencing decisions on tumor screening, initial treatment, and follow-up for NSCLC based on the advantages of minimal trauma, good repeatability, simplicity, and rapidity. TM can be used to assist diagnosis and differential diagnosis and to understand the possible pathological types of NSCLC. In the clinical diagnosis of NSCLC, carcinoembryonic antigen (CEA), cytokeratin 19 fragments antigen (CYFRA21-1), and squamous cell carcinoma antigen (SCC-Ag) have been used as reference TM for NSCLC. Other common TM also increased in NSCLC, including carbohydrate antigen 125 (CA125), CA15-3, CA19-9, and CA72-4. Previous studies have shown that TM level was correlated with pathological types, primary tissues [4], lymph node metastasis [5, 6], and distant metastasis [7]. Combination of TM can improve the diagnostic accuracy of NSCLC [8–10]. However, the points of view on combination of TM were not consistent. The correlation between TM and TNM stage of NSCLC has not been widely reported. Our research came from daily clinical practice. The TM levels came from before treatment since TM vary significantly before and after treatment [11, 12]. We chose benign chest diseases (BCD) that were easily confused with NSCLC as a control. We systematically discussed the characteristics of seven TM in pathological type, TNM stage, and diagnosis of NSCLC.

## 2. Materials and Methods

### 2.1. Patients

This study was approved by the ethics committee of the Guangxi Medical University Affiliated Tumor Hospital with an ethics approval number of LW2018011. When the patients suspected they had lung cancer, they were first admitted to the thoracic tumor surgery department. We systematically reviewed all patients who had been hospitalized in the thoracic tumor surgery department from October 2014 to August 2017. Cases with other malignancies and/or no pathology and/or no image were excluded. NSCLC and BCD were all first diagnosed. The diagnosis of NSCLC was established by histopathology. NSCLC was staged according to the 2009 seventh edition of the tumor-nodes-metastasis (TNM) classification for lung cancer [13–15]. The diagnosis of BCD was established by histopathology or pathogeny. BCD cases were not treated with any therapy. NSCLC cases had not been treated with any antitumor therapy, such as surgery, chemotherapy, radiotherapy, biological therapy, endocrine therapy, Chinese medicine treatment, hyperthermia, and radiofrequency ablation therapy. Complete and detailed case information were available for all cases. A total of 424 cases of lung adenocarcinoma (LAC), 166 cases of lung squamous cell carcinoma (LSCC), and 103 BCD patients were included in this study (Table 1).

### 2.2. Laboratory Assays

Each subject agreed to undergo fasting blood tests the next morning after admission, which was routine treatment. The test was completed on the day of blood drawing to avoid attenuation. All assays were performed according to instrument and reagent specifications. CEA, CA125, CA15-3, CA19-9, CA72-4, CYFRA21-1, and SCC-Ag were measured using ARCHITECTi2000_SR_ automatic electrochemical luminescence instrument and supporting reagents (Abbott Laboratories, Chicago). TM level above the following values was considered abnormal: CEA, 5 ng/ml; CA125, 35 ng/ml; CA15-3, 30 ng/ml; CA19-9, 37 ng/ml; CA72-4, 5.3 ng/ml; CYFRA 21-1, 3.3 ng/ml; and SCC-Ag, 1.5 ng/ml.

### 2.3. Statistical Analysis

IBM SPSS21.0 and MedCalc 18.2 software were applied for statistical analysis. The levels adopted for significance were *p* < 0.05  (two tailed). The calculation of sample content: *n* = *μ*_ɑ_^2^ p(1-p)/*δ* ^2^, the sample size of the case group and the control group were all 96, and the sample size of the study met the requirements. The normality test used skewness coefficient (*sk*) and kurtosis coefficient (*ku*). The TM data was not normal distribution. *Mann-Whitney U* test of nonparametric rank sum test was used to compare the median of two groups. Chi-square test (*χ* ^2^) was used to compare the positive rates of two groups. The correlation between TM level and TNM stage of NSCLC was analyzed by *Kendall's tau-b* correlation analysis. The levels of TM may be influenced by T stage, N stage, M stage, and other factors, so the correlation coefficient was low. In this paper, the correlation analysis was based on *p* < 0.05 not the correlation coefficient. With BCD as a control, binary logistic regression was used to calculate the hazard ratios of TM in NSCLC. Applying the receiver operating characteristic curve (ROC curve) to calculate the area under the curve (AUC) of TM in NSCLC, *z* test was used to compare AUC. Sensitivity, specificity, negative predictive value (NPV), and positive predictive value (PPV) were calculated.

## 3. Results

### 3.1. Patients' Characteristics

A total of 590 NSCLC patients and 103 BCD patients were included in the study. Table 1 summarizes patients' characteristics. Of note, the proportion of advanced NSCLC was higher than that of early NSCLC at initial diagnosis (Table 1).

### 3.2. Distribution Characteristics of Tumor Markers in Non-Small-Cell Lung Cancer and Benign Chest Disease

The positive rates of TM in BCD were CYFRA21-1 > CA72-4 > CA125 > CEA > CA19-9 > SCC-Ag > CA15-3. The median of TM in BCD was within normal range. The median and positive rates of CEA, CA125, CA15-3, CA19-9, CA72-4, and CYFRA21-1 in LAC were higher than those in BCD (*p* < 0.05). The median and positive rates of CEA, CA125, CYFRA21-1, and SCC-Ag in LSCC were higher than those in BCD (*p* < 0.05). Among them, the median and positive rates of CEA, CA125, and CYFRA21-1 in both LAC and LSCC were higher than those in BCD (*p* < 0.05). The median and positive rates of CEA, CA15-3, and CA19-9 in LAC were higher than those in LSCC (*p* < 0.05). The median and positive rates of CYFRA21-1 and SCC-Ag in LSCC were higher than those in LAC (*p* < 0.05) (Table 2).

### 3.3. Correlation Analysis of Tumor Markers and TNM Stage in Lung Adenocarcinoma and in Lung Squamous Cell Carcinoma

Through *Kendall's tau-b* correlation analysis, it was found that CEA, CA125, CA15-3, CA19-9, CA72-4, CYFRA21-1, and SCC-Ag were correlated with T stage of LAC (*r* = 0.235, *p* < 0.05; *r* = 0.298, *p* < 0.05; *r* = 0.254, *p* < 0.05; *r* = 0.063, *p* < 0.05; *r* = 0.080, *p* < 0.05; *r* = 0.268, *p* < 0.05; and *r* = 0.080, *p* < 0.05). CEA, CA125, CA15-3, CA19-9, CA72-4, and CYFRA21-1 were correlated with N stage of LAC (*r* = 0.356, *p* < 0.05; *r* = 0.415, *p* < 0.05; *r* = 0.340, *p* < 0.05; *r* = 0.117, *p* < 0.05; *r* = 0.175, *p* < 0.05; and *r* = 0.351, *p* < 0.05). CEA, CA125, CA15-3, CA19-9, CA72-4, and CYFRA21-1 were correlated with M stage of LAC (*r* = 0.365, *p* < 0.05; *r* = 0.353, *p* < 0.05; *r* = 0.293, *p* < 0.05; *r* = 0.135, *p* < 0.05; *r* = 0.169, *p* < 0.05; and *r* = 0.312, *p* < 0.05) (Table 3).

Through *Kendall's tau-b* correlation analysis, it was found that CA125, CYFRA21-1, and SCC-Ag were correlated with T stage of LSCC (*r* = 0.202, *p* < 0.05; *r* = 0.233, *p* < 0.05; and *r* = 0.099, *p* < 0.05). CA125 and CYFRA21-1 were correlated with N stage of LSCC (*r* = 0.178, *p* < 0.05 and *r* = 0.284, *p* < 0.05). CA125, CA15-3, and CYFRA21-1 were correlated with M stage of LSCC (*r* = 0.214, *p* < 0.05; *r* = 0.152, *p* < 0.05; and *r* = 0.213, *p* < 0.05) (Table 4).

### 3.4. Diagnostic Characteristics of Tumor Markers in Non-Small-Cell Lung Cancer

**Figure 1:**
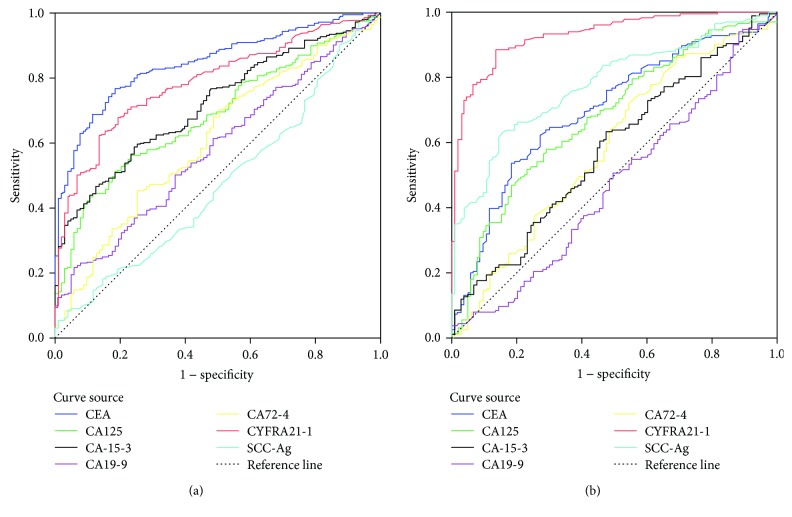
(a) ROC curve of tumor markers in lung adenocarcinoma. (b) ROC curve of tumor markers in lung squamous cell carcinoma.

The AUC of CEA, CA12-5, CA15-3, CA72-4, and CYFRA21-1 was statistically significant in LAC (all *p* < 0.05). The AUC of CEA in LAC was significantly higher than CA125 (*z* = 5.361, *p* < 0.05), CA15-3 (*z* = 4.629, *p* < 0.05), CA72-4 (*z* = 6.598, *p* < 0.05), and CYFRA21-1 (*z* = 2.162, *p* < 0.05). The AUC of CYFRA21-1 in LAC was significantly higher than CA12-5 (*z* = 3.147, *p* < 0.05), CA15-3 (*z* = 2.243, *p* < 0.05), and CA72-4 (*z* = 4.641, *p* < 0.05). The AUC of CEA, CA125, CYFRA21-1, and SCC-Ag was statistically significant in LSCC (all *p* < 0.05). The AUC of CYFRA21-1 in LSCC was significantly higher than CEA (*z* = 6.463, *p* < 0.05), CA125 (*z* = 7.112, *p* < 0.05), and SCC-Ag (*z* = 4.993, *p* < 0.05). The AUC of SCC-Ag in LSCC was significantly higher than CEA (*z* = 2.093, *p* < 0.05) and CA125 (*z* = 2.288, *p* < 0.05). Combining hazard ratio, AUC, sensitivity, specificity, NPV, and PPV, it was concluded that CEA and CYFRA21-1were the most related TM of LAC and SCC-Ag and CYFRA21-1 were the most related TM of LSCC (Table 5, Figure 1).

## 4. Discussions

TM was also expressed in BCD. Increased TM in BCD may be correlated with inflammation and disease severity [16]. Seven TM in BCD are out of the normal range to varying degrees. In this study, the parallel positive rate of seven TM in BCD was 41.7% and the tandem positive rate was 55.3%. The parallel positive rate of seven TM in LAC was 86.5% and the tandem positive rate was 245.3%. The parallel positive rate of seven TM in LSCC was 93.4% and the tandem positive rate was 212.1%. It was shown that TM in BCD mainly increased by one, and that in NSCLC mainly increased by two or more. The median of seven TM in BCD was within the normal range. The median of CEA and CYFRA21-1 in LAC and the median of CYFRA21-1 and SCC-Ag in LSCC had approached or exceeded the normal range. The median and positive rates of CEA, CA125, CA15-3, CA19-9, and CYFRA21-1 in LAC were higher than those in BCD (*p* < 0.05). The median and positive rates of CEA, CA125, CYFRA21-1, and SCC-Ag in LSCC were higher than those in BCD (*p* < 0.05). It was shown that TM was lowly expressed in BCD and highly expressed in NSCLC.

The expression of CEA in primary tissue and lymph node metastasis of LAC was higher than that in other NSCLC types [17]. CEA was highly expressed in nonlepidic dominant histologic subtype [18], adenocarcinoma tissues coexisting with bullae or honeycomb cysts [19] and well-differentiated adenocarcinoma tissues [20]. The expression of CEA was also correlated with TNM stage of NSCLC [21, 22]. CEA was correlated with T stage, N stage, and M stage of LAC (*r* = 0.235, *p* < 0.05, *r* = 0.356, *p* < 0.05, and *r* = 0.365, *p* < 0.05). It was shown that the level of CEA was correlated with primary tissue, lymphatic metastasis, and distant metastasis of LAC. However, CEA was not correlated with TNM stage of LSCC (*p* > 0.05). The correlation between CA19-9, CA72-4, and TNM stage in LAC and LSCC were similar to that of CEA; the correlation coefficients were lower. CA19-9 increased in patients with infectious lung disease [23]. NSCLC, especially advanced NSCLC, was often accompanied by severe infection. Therefore, CA19-9 can help identify NSCLC stage. In addition, CA19-9 was highly expressed in certain pathological types of LAC [24–26]. CA72-4 had not been reported in NSCLC.

CYFRA21-1 expressed in respiratory epithelial cells and has been detected in NSCLC tissue [27]. The increase of CYFRA21-1 in blood was more common in NSCLC, and its sensitivity was highest in LSCC and was considered as the best TM for LSCC [27, 28]. CYFRA21-1 was an independent predictor of NSCLC metastasis [7]. In this study, CYFRA21-1 was correlated with T stage, N stage, and M stage of both LAC and LSCC (*r* = 0.268, *p* < 0.05; *r* = 0.351, *p* < 0.05; *r* = 0.312, *p* < 0.05; *r* = 0.233, *p* < 0.05; *r* = 0.284, *p* < 0.05; and *r* = 0.231, *p* < 0.05). CYFRA21-1 was risk index for both LAC and LSCC (*p* < 0.05). In LAC, the AUC of CYFRA21-1 (0.79) was second only to CEA (0.85). In LSCC, the AUC of CYFRA21-1 was the largest (0.93). Therefore, CYFRA21-1 was a more reliable TM for the diagnosis of NSCLC. The level of CYFRA21-1 was correlated with primary tissue, lymphatic metastasis, and distant metastasis of NSCLC. The expression of CA125 was not related to the degree of malignancy [29] but related to the degree of tissue differentiation. CA125 was highly expressed in less-differentiated tumor [20]. CA125 was positively correlated with postoperative metastasis [5] and postoperative recurrence in NSCLC [30]. Similar to CYFRA21-1, CA125 was correlated with T stage, N stage, and M stage of both LAC and LSCC (*r* = 0.298, *p* < 0.05; *r* = 0.415, *p* < 0.05; *r* = 0.353, *p* < 0.05; *r* = 0.298, *p* < 0.05; *r* = 0.415, *p* < 0.05; and *r* = 0.353, *p* < 0.05). The expression CA125 in NSCLC was also correlated with primary tissue, lymphatic metastasis, and distant metastasis.

The expression of SCC-Ag was correlated with differentiation degree of squamous cell carcinoma tissue. SCC-Ag was highly expressed in well-differentiated squamous cell carcinoma tissues and lowly expressed in poorly differentiated squamous cell carcinoma tissues [31]. The level of SCC-Ag was correlated with T stage of both LAC and LSCC (*r* = 0.080, *p* < 0.05; *r* = 0.099, *p* < 0.05). The level of SCC-Ag may be correlated with the highly differentiated squamous cell carcinoma of the lung. Due to the low sensitivity, SCC-Ag provided no additional value when used in combination with CYFRA21-1 to diagnose NSCLC [32]. Compared with CYFRA21-1, SCC-Ag has lower sensitivity (39.8%) but higher specificity (96.1%) for LSCC. SCC-Ag was still a widely used TM to identify pathological types of NSCLC and still an effective method for the identification of benign and malignant solitary pulmonary nodules [33].

CA15-3 was a soluble form of mucin-1 serum shedding [34]. Mucin-1 expression may be associated with nonsquamous cell carcinoma tissue [35]. CA15-3 level was correlated with T stage, N stage, and M stage of LAC (*r* = 0.254, *p* < 0.05; *r* = 0.340, *p* < 0.05; and *r* = 0.293, *p* < 0.05). CA15-3 was also correlated with M stage of LSCC (*r* = 0.168, *p* < 0.05). CA15-3 in LAC may be correlated with primary tissue, lymph node metastasis, and distant metastasis. CA15-3 in LSCC may indicate distant metastasis. However, Liu et al. [36] reported that mucin-1 mRNA in NSCLC peripheral blood cannot be used as a reliable TM of metastasis. The value of CA15-3 in diagnosis of NSCLC still needs further study.

## 5. Conclusion

CEA combined with CYFRA21-1 contributed to auxiliary diagnosis of LAC. CYFRA21-1 combined with SCC-Ag contributed to auxiliary diagnosis of LSCC. CEA, CA125, CA15-3, CA19-9, CA72-4, and CYFRA21-1 were correlated with primary tissue and metastasis of LAC. CA125 and CYFRA21-1 were correlated with primary tissue and metastasis of LSCC.

## Figures and Tables

**Table 1:** Characteristics of the study subjects.

Parameters	NSCLC *n* = 590	Parameters	BCD *n* = 103
Gender		Gender	
Male	402	Male	55
Female	188	Female	48
Age (year)		Age (year)	
Range	28-86	Range	18-76
Mean	57.7	Mean	53.9
Pathological type		Disease	
LAC	424	Hemangioma	4
LSCC	166	Hamartoma	6
T stage		Inflammatory pseudotumor	8
T1	119	Fibrous nodules	6
T2	260	Tuberculosis	22
T3	176	Infection	33
T4	35	Lymphoproliferation	2
N stage		Cysts	12
N0	172	Thymoma	3
N1	65	Substernal thyroid	1
N2	222	Amyloidosis	1
N3	131	Lipoma	1
M stage		Schwannoma	4
M0	376	—	—
M1	214	—	—

BCD: benign chest disease; NSCLC: non-small-cell lung cancer; LAC: lung adenocarcinoma; LSCC: lung squamous cell carcinoma.

**Table 2:** The median and positive rates of tumor markers in benign chest disease (BCD), lung adenocarcinoma (LAC), and lung squamous cell carcinoma (LSCC).

Parameters	BCD *n* = 103	LAC *n* = 424	LSCC *n* = 166	*p*1 value	*p*2 value	*p*3 value
Median (ng/ml)						
CEA	2.1	7.4	3.3	0.000	0.000	0.000
CA125	12.8	22.5	20.0	0.000	0.000	0.074
CA15-3	12.3	20.4	15.0	0.000	0.035	0.000
CA19-9	10.5	14.0	10.6	0.010	0.397	0.000
CA72-4	1.3	2.0	1.8	0.000	0.019	0.164
CYFRA21-1	2.3	4.1	7.6	0.000	0.000	0.000
SCC-Ag	0.7	0.6	1.3	0.443	0.000	0.000
Positive rate (%)						
CEA	7.8	61.8	23.5	0.000	0.001	0.000
CA125	8.7	40.6	28.9	0.000	0.000	0.009
CA15-3	2.9	30.2	9.0	0.000	0.051	0.000
CA19-9	5.8	19.8	5.4	0.001	0.888	0.000
CA72-4	11.7	21.7	16.9	0.022	0.242	0.190
CYFRA21-1	13.6	61.6	88.6	0.000	0.000	0.000
SCC-Ag	3.9	8.5	39.8	0.113	0.000	0.000

*p 1* value: BCD vs LAC, *p2* value: BCD vs LSCC, *p3* value: LAC vs LSCC.

**Table 3:** Correlation analysis of tumor markers and TNM stage in lung adenocarcinoma.

Parameters	T stage		N stage		M stage	
	*r*	*p* value	*r*	*p* value	*r*	*p* value
CEA	0.235	0.000	0.356	0.000	0.365	0.000
CA125	0.298	0.000	0.415	0.000	0.353	0.000
CA15-3	0.254	0.000	0.340	0.000	0.293	0.000
CA19-9	0.063	0.045	0.117	0.001	0.135	0.000
CA72-4	0.080	0.015	0.175	0.000	0.169	0.000
CYFRA21-1	0.268	0.000	0.351	0.000	0.312	0.000
SCC-Ag	0.080	0.016	0.000	0.497	−0.028	0.245

**Table 4:** Correlation analysis of tumor markers and TNM stage in lung squamous cell carcinoma.

Parameters	T stage		N stage		M stage	
	*r*	*p* value	r	*p* value	*r*	*p* value
CEA	0.078	0.092	0.038	0.263	0.020	0.379
CA125	0.202	0.000	0.178	0.001	0.214	0.000
CA15–3	−0.045	0.222	0.013	0.416	0.152	0.008
CA19-9	0.044	0.227	0.029	0.312	0.047	0.230
CA72-4	0.061	0.152	0.051	0.193	−0.013	0.419
CYFRA21-1	0.233	0.000	0.284	0.000	0.213	0.000
SCC-Ag	0.099	0.046	0.046	0.217	0.035	0.290

**Table 5:** The diagnostic characteristics of tumor markers in lung adenocarcinoma (LAC) and lung squamous cell carcinoma (LSCC).

Parameters	Hazard ratio	AUC	Sensitivity	Specificity	NPV	PPV
LAC (*n* = 424)						
CEA	1.34^∗^	0.85^∗^	61.8	92.2	37.0	97.0
CA125	1.00	0.69^∗^	40.6	91.3	27.2	95.0
CA15-3	1.03	0.72^∗^	30.2	97.1	25.3	97.7
CA19-9	1.00	0.58	19.8	94.2	22.2	93.3
CA 72-4	1.01	0.61^∗^	21.7	88.3	21.5	88.5
CYFRA21-1	1.43^∗^	0.79^∗^	61.6	86.4	35.3	94.9
SCC-Ag	1.03	0.48	8.5	96.1	20.3	90.0
LSCC (*n* = 166)						
CEA	1.09	0.70^∗^	23.5	92.2	42.8	83.0
CA125	1.00	0.68^∗^	28.9	91.3	44.3	84.2
CA15-3	1.03	0.58	9.0	97.1	39.8	83.3
CA19-9	1.00	0.47	5.4	94.2	38.2	60.0
CA72-4	1.03	0.59	16.9	88.3	39.7	70.0
CYFRA21-1	2.26^∗^	0.93^∗^	88.6	86.4	82.4	91.3
SCC-Ag	2.28^∗^	0.78^∗^	39.8	96.1	49.7	94.3

^∗^ *p* value < 0.05.

## Data Availability

The data availability statement is listed in the manuscript.

## Supplementary Materials

Supplementary Materials Supplemental material is raw data. The non-small-cell lung cancer sheet includes gender, age, pathological type (1 = lung adenocarcinoma and 2 = lung squamous cell carcinoma), tumor marker levels (CEA, CA125, CA15-3, CA19-9, CA72-4, CYFRA211, and SCC-Ag), and TNM stage. The benign chest disease sheet includes gender, age, tumor marker levels (CEA, CA125, CA15-3, CA19-9, CA72-4, CYFRA211, and SCC-Ag), and diagnostic classification.
